# Effects of Prenatal Methamphetamine Exposure on the Developing Human Brain: A Systematic Review of Neuroimaging Studies

**DOI:** 10.1021/acschemneuro.1c00213

**Published:** 2021-07-23

**Authors:** Hossein Sanjari Moghaddam, Maryam Mobarak Abadi, Mahsa Dolatshahi, Sasan Bayani Ershadi, Fatemeh Abbasi-Feijani, Sahar Rezaei, Giulia Cattarinussi, Mohammad Hadi Aarabi

**Affiliations:** † Faculty of Medicine, Tehran University of Medical Sciences, Tehran, Iran; ‡ Faculty of Medicine, Arak University of Medical Sciences, Arak, Iran; § Department of Neuroscience and Padova Neuroscience Center (PNC), University of Padova, 35131 Padova, Italy

**Keywords:** Prenatal methamphetamine, neuroimaging, neurodevelopmental disorder, MRI

## Abstract

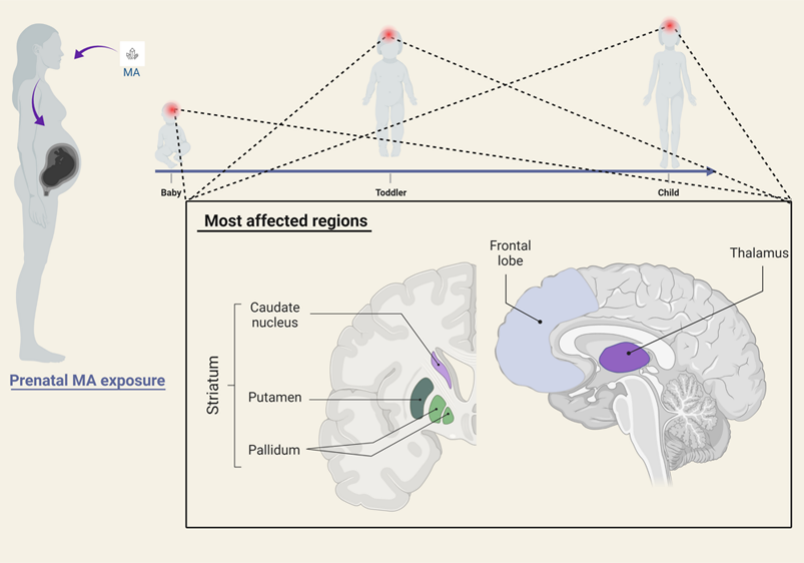

Methamphetamine (MA) can cross the placenta in pregnant women and cause placental abruption and developmental alterations in offspring. Previous studies have found prenatal MA exposure effects on the social and cognitive performance of children. Recent studies reported some alterations in structural and functional magnetic resonance imaging (MRI) of prenatal MA-exposed offspring. In this study, we aimed to investigate the effect of prenatal MA exposure on brain development using recently published structural, metabolic, and functional MRI studies. According to the Preferred Reporting Items for Systematic Reviews and Meta-Analyses (PRISMA) guidelines, we searched PubMed and SCOPUS databases for articles that used each brain imaging modality in prenatal MA-exposed children. Seventeen studies were included in this study. We investigated brain imaging alterations using 17 articles with four different modalities, including structural MRI, diffusion tensor imaging (DTI), magnetic resonance spectroscopy (MRS), and functional MRI (fMRI). The participants’ age range was from infancy to 15 years. Our findings demonstrated that prenatal MA exposure is associated with macrostructural, microstructural, metabolic, and functional deficits in both cortical and subcortical areas. However, the most affected regions were the striatum, frontal lobe, thalamus and the limbic system, and white matter (WM) fibers connecting these regions. The findings from our study might have valuable implications for targeted treatment of neurocognitive and behavioral deficits in children with prenatal MA exposure. Even so, our results should be interpreted cautiously due to the heterogeneity of the included studies in terms of study populations and methods of analysis.

## Introduction

Methamphetamine (MA), contracted from *N*-methylamphetamine, is a potent psychostimulant that belongs to the substituted phenethylamine and substituted amphetamine chemical classes, many of which are formed by a phenyl ring connected to an amino group by a two-carbon side chain.^1^ It targets the dopamine transporter (DAT) in the brain and thus increases extracellular dopamine and alters neuronal activity in the reward system.^2^ Moreover, it has indirect agonist properties on serotonin and noradrenaline receptors, alters glutamate^3^ and GABA^4^ brain levels, and inhibits some neurotransmitter degradations.^5^ It can be used via oral, nasal, rectal, or intravenous routes. Its acute consumption can cause euphoria, high energy levels, and alertness, along with an increase in libido and sexual pleasure.^6,7^ MA is abused as a highly addictive stimulant in various populations from young men to pregnant women.^8^ The number of pregnant women abusing MA increased over the past decades, and its effects on neonates are not still completely known.^9^ Epidemiological investigations have shown that primary caretakers of MA-exposed neonates may have lower education and socioeconomic status and higher rates of unemployment. Moreover, simultaneous usage of alcohol, nicotine, and marijuana makes it hard to investigate specific MA-related alterations.^10,11^

MA can cross the placenta and cause placental inefficiency and abruption, deterioration of intrauterine growth, and preterm birth.^12,13^ Previous studies have found detrimental effects of prenatal MA exposure on children. In particular, offspring can suffer from withdrawal syndrome at birth and a significant number of complications as they grow up, including increased cognitive impairments, stress, lethargy, and difficulties in executive function and working memory accompanied by poor motor skills and psychomotor adjustment.^14−16^

Recent studies highlighted probable brain alterations in children associated with prenatal MA exposure.^11,17,18^ Structural magnetic resonance imaging (MRI), diffusion tensor imaging (DTI), magnetic resonance spectroscopy (MRS), and functional MRI (fMRI) have shown a range of macrostructural, microstructural, metabolic, and functional changes associated with prenatal MA exposure. Morphologic and structural alterations can be detected with structural MRI studies. Differently, DTI describes alterations in brain microstructure employing different aspects of water molecule diffusion. For instance, water diffusion in a tissue is restricted due to some local structures, such as the cell membrane and myelin sheath, causing unequal diffusion in different directions or anisotropy. On the basis of the patterns of water diffusion and the extent of diffusion restriction in some directions, the orientation and microstructural features of white matter (WM) fibers within a voxel can be determined.^19^ Fractional anisotropy (FA) is a measure of anisotropy that depends on the number and density of axons in a voxel and represents the microstructural coherence of WM fibers.^20^ The FA value is sensitive to any change in extracellular or intracellular liquid content, inflammation, axonal loss, gliosis, and demyelination, most of which present with a decreased FA; mean diffusivity (MD) measures diffusion of water in all directions within a voxel. Thus, increased MD represents the freedom of diffusion as a result of increased extracellular spaces due to axonal degeneration or demyelination; axial and radial diffusivity (AD and RD) are measures of water diffusivity parallel and perpendicular to WM tracts, respectively, where increased AD represents axonal degeneration and increased RD reflects demyelination.^21−23^ MRS provides valuable insight into the biochemistry of different brain regions by assessing several markers of neuronal and glial integrity, such as N-acetylaspartate, creatine, choline, glutamine, and glutamate.^24^ Finally, fMRI assesses the alterations in brain activity or connectivity by employing the blood-oxygen level dependent (BOLD) signal as a proxy of neural function.^18,25^

A better understanding of the structural, microstructural, metabolic, and functional abnormalities in offspring associated with prenatal MA exposure might contribute to the development of more effective interventions for cognitive and behavioral deficits in children exposed to prenatal MA use. In this study, we aimed to investigate the effect of prenatal MA exposure on brain development using recently published structural, metabolic, and functional MRI studies.

## Methods

We performed this systematic Review under the Preferred Reporting Items for Systematic Reviews and Meta-Analyses (PRISMA) guidelines.^26^

### Literature Search and Selection Criteria

To identify relevant studies, PubMed and SCOPUS were searched for relevant articles, published between the earliest record and June 1, 2021. The search terms included “Prenatal exposure OR Perinatal OR Pregnancy OR Maternal” AND “Diffusion Tensor Magnetic Resonance Imaging OR Diffusion Tensor Imaging OR Functional Magnetic Resonance Imaging OR Neuroimaging” AND “Methamphetamine OR Methylamphetamine OR Deoxy ephedrine OR Hydrochloride” and equivalent terms in each database. We also checked for additional eligible studies by going through the reference list of the relevant articles.

**Figure 1:**
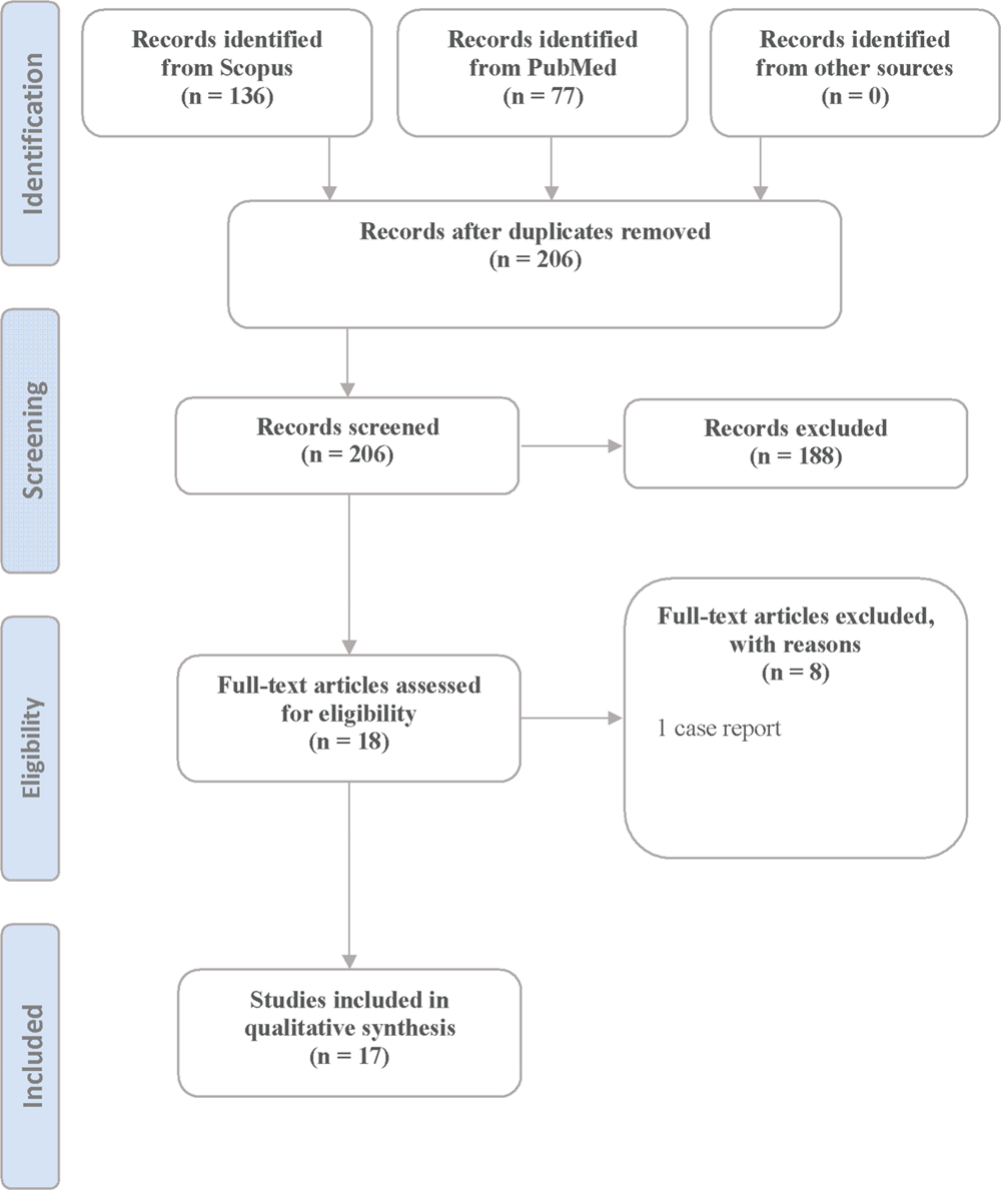
PRISMA flow diagram for neuroimaging studies in prenatal methamphetamine (MA) exposure.

Studies were included if they (1) measured brain structure or function using structural MRI, DTI, H-MRS, and fMRI, (2) compared children born from mothers using MA with age- and gender-matched children born from healthy nonusing mothers, (3) were original peer-reviewed studies, and (4) were in English. Studies on women with severe medical or psychiatric comorbidities and/or women on medications were excluded. We excluded case reports, case series, letters, commentaries, abstracts, review articles, and in vivo and in vitro studies. Data selection was in concordance with the PRISMA guidelines.^27^ Two authors (M.M.A. and M.H.A.) independently performed the eligibility assessment. In the case of disagreement, the two authors discussed and resolved the conflict, and if they could not reach an agreement, a third person intervened to make the final decision. The PRISMA chart for this study is provided in Figure 1.

### Data Extraction

Two data extraction tables were designed to extract the relevant information on studies included in this Review (Tables 1 and 2). Data extraction was performed by one author and checked by another author. If there was any disagreement, a third person was asked to finalize the decision. The extracted demographic and social/habitual details of the mothers and offspring included age, sex, gestational age, head circumference, and special characteristics or exclusion criteria of offspring and age, education, depression, and amount and frequency of methamphetamine, alcohol, nicotine/tobacco, and marijuana use of the mothers during pregnancy. Type of brain imaging, analysis method and toolbox, key imaging findings, and cognitive and behavioral characteristics of the children were extracted in Table 2.

**Table 1:** Overview of Reviewed Articles: Demographic Profile of Offspring and the Related Maternal Profile by Imaging Modality^a^

			offspring profile				maternal profile								
										drug use during pregnancy					
	ref	study groups^b^	*N*/male	*A*_scan_^c^	*A*_gest_^d^	*C*_head_^e^ at birth	*A*_deliv_^f^	years of educ.^g^	depr.^h^	MA	alc	nic/tob	marij	matched in	differed in
Structural MRI Studies															
1	(17)	MA-exposed	13/4	6.9 ± 3.5	–	–	–	–	–	–	–	–	–	age, gender distrib, both recruited from the same population of predominately lower and middle soc/ec status	–
		unexposed	15/6	7.8 ± 3.2											
		(inclusion based on DSM-IV criteria for at least two-thirds of the pregnancy; exclusion criteria: GA of less than 37 weeks, developmental delay, impaired growth, seizure disorders or ADHD, significant maternal illness during gestation, dependence on other illicit drugs (except Meth) or alcohol)		(years)											
2	(32)	MA-exposed	21/12	9.66 ± 1.85	–	–	–	–	–	3/3 of interviewd mothers reported use of MA/alc during pregnancy, the rest suffered social/legal problems	18/21 with alc exposure, 3/3 of interviewd mothers reported use of MA/alc during pregnancy, the rest suffered social/legal problems	–	–	age, gender distrib, handedness, soc/ec status (family annual income)	FSIQ (*p* < 0.001) with the unexposed group scoring significantly higher than the MA-exposed and alc-exposed groups
		alcohol-exposed	13/8	11.15 ± 2.34											
		unexposed	27/11	10.15 ± 2.90											
		(exclusion criteria: children with prenatal exposure to cocaine or other opiates, who were <5 years of age, who had an IQ < 70, or had a physical, psychiatric, or developmental disability, or any other potential known causes of mental deficiency)		(years)											
3	(33)	MA-exposed	20/11	45.76 ± 6.78	38.55 ± 1.85	–	25.5 ± 6.7	10	6.81 ± 7.46	19/26 used MA	–	19/20 used tob	–	age, gender distrib, handedness, *A*_gest_, maternal age, maternal education, birth weight, prenatal care, maternal depression or psychological distress over the first 3 years of the study	between tob-exposed and tob-unexposed groups: prenatal exposure to MA (*p* = 0.002); between MA-exposed and MA-unexposed groups: prenatal exposure to tob (*p* = 0.002), prenatal marij (*p* = 0.027), *C*_head_ (*p* = 0.011), higher (worse) HRT by ISI scores (*p* = 0.002), and borderline higher HRT by block scores (*p* = 0.06)
		not MA-exposed	15/8	47.26 ± 7.88	39.20 ± 1.15		22.3 ± 3.0	8	8.76 ± 6.45	1/9 used MA		7/15 used tob			
		tobacco-exposed	26/19	46.43 ± 6.85	38.58 ± 1.65		24.2 ± 5.5	15	6.92 ± 6.97						
		not tobacco-exposed	9/9	46.33 ± 8.59	39.56 ± 1.24		23.9 ± 6.2	3 (number of participants with less than a high school education)	9.74 ± 7.11						
		(maternal exclusion criteria: not speaking English; <18 years of age; used opiates, LSD, PCP, or cocaine only during pregnancy; severe retardation, emotional, cognitive, or past psychosis; infant exclusions: critically ill and unlikely to survive; multiple gestation; life-threatening congenital anomaly; chromosomal abnormality associated with mental or neurological deficiency; overt clinical evidence of an intrauterine infection, or sibling previously enrolled in the study)		(months)											
4	(34)	MA-exposed	18/10	6.45 ± 0.42	–	51.75 ± 1.72	–	8.88 ± 1.32	–	–	3 mothers in the MA-exposed group	–	–	age, gender distrib, soc/ec profile, gestation, birth circumstances, income, and schooling	education (*p* = 0.044), unemployment rate (76% vs 44%)
		unexposed	18/8	6.51 ± 0.33		51.99 ± 2.41		10.25 ± 2.32							
		(exclusion criteria: fetal anomalies; history of epilepsy, diabetes, head injury, or prematurity); three of the mothers in the MA-exposed group also used alcohol; mothers/caregivers stated that no other drugs (e.g., cocaine, heroin) were used besides MA during the pregnancy; none of the mothers of the controls used alcohol or illicit drugs		(years)		(current *C*_head_)									
5	(29)	MA-exposed	18/6	40.5 ± 2.1	37.6 ± 2.7	32.9 ± 1.9	27.0 ± 4.2	9.3 ± 1.4	–	–	10/18: 0.2 ± 0.4 oz AA/day	18/18: 6.5 ± 4.5 cig/day	10/18: 5.8 ± 8.4 days/month	age, gender distrib, maternal age, *A*_gest_, *C*_head_, food security, and maternal weight gain	abusing tob (*p* = 0.021), alc (*p* = 0.038), and marij (*p* = 0.003), maternal education (*p* = 0.013)
		unexposed	21/12	41.6 ± 1.9	39.1 ± 2.0	33.3 ± 2.0	26.7 ± 5.9	10.5 ± 1.4			1/21: 0.0 ± 0.0 oz AA/day	13/21: 3.5 ± 3.4 cig/day	2/21: 0.0 ± 0.1 days/month		
		(the control group, recruited from the same community, comprising infants whose mothers abstained from alcohol and other drugs of abuse or who consumed no more than 2 drinks on 2 or fewer occasions during pregnancy; exclusion criteria for mothers were <18 years of age, HIV positive, treatment for medical conditions such as hypertension, epilepsy, diabetes or heart disease, and multiple pregnancies; infant exclusion criteria were neural tube defects, major chromosomal anomalies, very low birth weight (<1200 g), gestational age <30 weeks, and seizures)		(*A*_gest_ in weeks) between 1 and 4 weeks after birth, with the exception of two infants born prior to 34 weeks who were scanned at 7 and 9 weeks of age, respectively											
6	(38)	MA-exposed	17/10	6.9 ± 0.4^i^				9 ± 1.3			4/17 consuming alc	13/17 smoking		age, gender distrib, maternal educ	cig smoking (*p* = 0.024), mother as the primary caregiver (*p* = 0.002)
				8.0 ± 0.5^j^											
		unexposed	16/7	6.5 ± 0.3^i^				10 ± 2.2				6/16 smoking			
				8.3 ± 0.4^j^											
		(exclusion: history of genetic anomalies, neurological disorders, head injury, or prematurity)		(years)											
DTI Studies															
1	(11)	MA-exposed	29/20	48.4 ± 1.4	38.8 ± 0.3	50.5 ± 0.3	25.1 ± 1.1	12.7 ± 0.4	–	2.5 ± 0.2 trimesters, 57.9 ± 31.0 g (data for 16 mothers)	15.3 ± 9.8 drinks (data for 15 mothers)	88 ± 26 packs (data for 20 mothers)	18.2 ± 17.0 joints (data for 16 mothers)	age, gender distrib, *A*_gest_ at birth, parental estimated verbal intelligence, current *C*_head_, and BMI	lower maternal/caretaker educ, index of social position (*p* = 0.05), nic exposure (*p* = 0.04), alc exposure (*p* = 0.02), birth weight (*p* = 0.002), maternal age at birth (*p* = 0.008)
		unexposed	37/19	47.7 ± 1.2	39.2 ± 0.2	50.5 ± 0.2	29.9 ± 1.3	14.2 ± 0.5		NA	0.1 ± 0.01 drinks	25 ± 16 packs	0.8 ± 0.8 joints		
		(exclusion criteria: any congenital, genetic, or other major neurological disorders, prematurity, FTT within the first year of life, overt TORCH infection at birth, mother or legal guardian who were non-English speaking, low cognitive functioning (estimated verbal IQ < 80), HIV-1 during pregnancy, a history of comorbid psychiatric illness or a major medical condition compromising brain development, a history of DSM-IV dependence on alcohol or other drugs except nicotine and MA during pregnancy)		(months)		(current *C*_head_)									
2	(30)	MA-exposed	21/13	9.76 ± 1.84	–	–	–	15.13 ± 2.29	–	–	–	12/21 exposed, 1/21 not exposed, 8/21 unknown	–	age, gender distrib, handedness, parental educ, parental IQ, family income, trails B performance, number of scan averages	prorated full-scale IQ (*p* < 0.05), parent type (adopted vs biological) (*p* < 0.001), nic exposure (*p* < 0.001), visuomotor integration (*p* < 0.005)
		unexposed	27/11	10.30 ± 3.35				16.33 ± 2.87				27/27 not exposed			
		(exclusion criteria: age younger than 5 years, IQ < 70, head injury with loss of consciousness for >20 min, other causes of mental deficiency like chromosomal syndromes, any physical or psychiatric illness, or developmental disability preventing completion of the scanning or neuropsychological testing sessions)		(years)											
3	(10)	MA-exposed	17/9	6.71 ± 0.40	–	51.79 ± 1.76	–	8.81 ± 1.33	–	–	3/17 consuming alc	14/17 smokers	–	age, gender distrib, current weight, length, and *C*_head_, maternal educ	smoking (*p* = 0.02)
		unexposed	15/5	6.83 ± 0.39		51.86 ± 1.79		9.93 ± 2.30				6/15 smokers			
		(exclusion: history of genetic anomalies, neurological disorders, head injury, or prematurity)		(years)		(current *C*_head_)									
4	(28)	MA/tobacco-exposed	36/19	57.21 ± 8.05	39.04 ± 0.28	34.07 ± 0.22	28.36 ± 1.12	11.71 ± 0.32	BDI: 11.94 ± 1.71	–	15/36 (0.56 ± 0.13 trimesters, 7.92 ± 2.94 drinks)	35/36 (2.28 ± 0.15 trimesters, 1364 ± 341 cig)	15/36 (0.78 ± 0.18 trimesters, 36.71 ± 31.97 cig)	age, *A*_gest_, race, *C*_head_ and weight at birth, maternal age at delivery	sex (*p* = 0.03), marij use and exposure (*p* < 0.001), alc use (*p* = 0.02) and no. of drinks (*p* = 0.002), tob use (*p* < 0.001), soc/ec status (*p* < 0.001), maternal educ (*p* < 0.001), weight gain during pregnancy (p < 0.001), BDI scores (*p* = 0.04), weight (*p* = 0.003), height (*p* = 0.03), and *C*_head_ (*p* = 0.03) at scanning time, neurologic examinations (*p* = 0.009)
									EDPS: 5.44 ± 0.68						
		tobacco-exposed	32/21	56.44 ± 6.94	37.47 ± 0.69	32.85 ± 0.74	27.50 ± 0.91	12.50 ± 0.30	BDI: 9.91 ± 1.40		10/32 (0.34 ± 0.09 trimesters/2.78 ± 0.96 drinks)	32/32 (1.78 ± 0.16 trimesters/2717 ± 407 cig)	7/32 (0.31 ± 0.11 trimesters/32.41 ± 18.84 cig)		
									EPDS: 7.40 ± 0.93						
		unexposed	71/27		38.39 ± 0.36	33.90 ± 0.36	28.89 ± 0.68	14.28 ± 0.30	BDI: 7.48 ± 0.91		13/71 (0.27 ± 0.07 trimesters/0.83 ± 0.38 drinks)	0/71	1/71 (0.01 ± 0.01 trimesters/0.03 ± 0.03 cig)		
									EPDS: 7.85 ± 1.07						
		(exclusion criteria: infants with excessive maternal alcohol use, maternal polysubstance or cocaine dependency, human immunodeficiency virus-infected mother receiving zidovudine or prolonged neonatal intensive care)		(weeks)											
5	(31)	MA-exposed	11/5	40.6 ± 2.1	37.3 ± 3.0	–	27.2 ± 3.9	9.4 ± 1.2	–	7.1 ± 3.5 days/month	0.2 ± 0.4 oz AA/day	6.5 ± 5.4 cig/day	4.4 ± 9.6 days/month	age, gender distrib, *A*_gest_ at birth, birth weight, maternal age, no difference in other substance use	maternal educ (*p* = 0.018)
		unexposed	12/7	41.6 ± 1.9	39.0 ± 1.7		25.1 ± 5.4	10.6 ± 1.1		NA	0.0 ± 0.0 oz AA/day	3.3 ± 2.8 cig/day	0.0 ± 0.1 days/month		
				(*A*_gest_/weeks)											
6	(61)	MA-exposed	11/5	40.6 ± 2.1	37.3 ± 3.0		27.2 ± 3.9	9.4 ± 1.2		7.1 ± 3.5 days/month	0.2 ± 0.4 oz AA/day	6.5 ± 5.4 cig/day	4.4 ± 9.6 days/month	age, gender distrib, *A*_gest_ at birth, birth weight, maternal age, no difference in other substance use	maternal educ (*p* = 0.018)
		unexposed	12/7	41.6 ± 1.9	39.0 ± 1.7		25.1 ± 5.4	10.6 ± 1.1		NA	0.0 ± 0.0 oz AA/day	3.3 ± 2.8 cig/day	0.0 ± 0.1 days/month		
		(exclusion: maternal age <18 years, multiple gestation pregnancy, HIV positive, or receiving treatment for medical conditions, including hypertension, heart disease, epilepsy, or diabetes; infants with neural tube defects, seizures, and chromosomal abnormalities)		(*A*_gest_/weeks)											
MRS Studies															
1	(24)	MA-exposed	12/?	8.1 ± 0.8	–	–	–	–	–	2/12 reported “trying” cocaine, occasionally	4/12 reported alc use (all <0.5 oz. of AA/day)	6/12: 17 ± 8 cig/day	–	–	–
		unexposed	14/?	7.3 ± 1.1							0/14 exposed	1/14 exposed			
		(exclusion criteria: preterm infants, developmental delay, impaired growth, seizure disorders, or ADHD, major maternal illness or requiring medication for a chronic illness, other illicit drug abuse)		(years)											
2	(35)	MA-exposed	49/30	46.90 ± 1.07	38.74 ± 0.23	50.37 ± 0.21	24.53 ± 0.99	12.19 ± 0.30	BDI: 10.41 ± 2.05	2.42 ± 0.14 trimesters	0.85 ± 0.21 trimesters	2.05 ± 0.21 trimesters	1.00 ± 0.22 trimesters	age, gender distrib, current height, current weight, current *C*_head_, birth length, maternal age at birth, maternal BDI depression score, maternal verbal IQ, primary care provider’s educ, soc/ec status	alc use (*p* = 0.002), nic use (*p* < 0.001), marij use (*p* < 0.001), birth weight (*p* = 0.003), *A*_gest_ at birth (*p* = 0.01), maternal educ (*p* = 0.082)
		unexposed	49/27	45.17 ± 0.99	39.47 ± 0.18	50.63 ± 0.18	26.06 ± 0.96	12.92 ± 0.29	BDI: 10.53 ± 1.75	NA	0.21 ± 0.08 trimesters	0.67 ± 0.17 trimesters	0.12 ± 0.07 trimesters		
		(exclusion criteria were any congenital, genetic, or other major neurological disorders, prematurity, FTT within the first year of life, overt TORCH infection at birth, mothers with age <17 years, non-English speaking, low cognitive functioning (estimated verbal IQ < 80) or institutionalized for retardation, HIV-1 during pregnancy, a history of comorbid psychiatric illness or a major medical condition compromising brain development, a history of drug dependence during pregnancy except for MA and nicotine)		(months)		(current *C*_head_)					(in the MA-exposed group, light alc use, i.e., less than 1 drink/day, was allowed)		(in the MA-exposed group, light marij usewas allowed)		
fMRI Studies															
1	(36)	MA-exposed	14/10	9.5 ± 1.91	–	–	–	15.93 ± 2.84	–	–	12/14 prenatal alc exposure (comparable to alc-exposed groups)	6/14 exposed, 7/14 unknown exposure	–	age, gender distrib, handedness, parental educ, parental IQ, family annual income	FSIQ (*p* = 0.01), adopted (*p* < 0.01), nic exposure (*p* < 0.01)
		unexposed	20/9	10.5 ± 2.56				15.80 ± 3.19				0/20 exposed to nic			
		(exclusion criteria: concomitant diagnosis of FAS or PFAS (severe manifestations of heavy alcohol exposure), known prenatal exposure to cocaine or opiates, age less than 7 years, IQ less than 70, head injury with loss of consciousness over 20 min, physical, psychiatric, or developmental disability, other known causes of mental deficiency like chromosomal anomalies, major maternal illness compromising brain development)													
2	(18)	MA-exposed	19/11	9.16 ± 1.83	–	–	–	–	–	–	–	–	–	age, gender distrib	FSIQ (*p* = 0.017), accuracy on the N-Back task (*p* = 0.002), accuracy on 2-Back condition of the task (*p* = 0.019)
		unexposed	18/9	10.28 ± 2.61											
		(exclusion criteria: known prenatal exposure to cocaine or opiates, age less than 7 years, IQ less than 70, head injury with loss of consciousness over 20 min, physical, psychiatric, or developmental disability, other known causes of mental deficiency like chromosomal anomalies, major maternal illness compromising brain development, performing below 1.5 standard deviations from the mean performance of their group on the N-Back task, or poor fMRI data quality													
3	(25)	MA-exposed	19/11	9.16 ± 1.83	–	–	–	–	–	–	–	–	–	age, gender distrib	FSIQ (*p* = 0.017), accuracy on the N-Back task (*p* = 0.002), accuracy on 2-Back condition of the task (*p* = 0.019)
		unexposed	18/9	10.28 ± 2.61											
		(exclusion criteria: known prenatal exposure to cocaine or opiates, age less than 7 years, IQ less than 70, head injury with loss of consciousness over 20 min, physical, psychiatric, or developmental disability, other known causes of mental deficiency like chromosomal anomalies, major maternal illness compromising brain development, performing below 1.5 standard deviations from the mean performance of their group on the N-Back task, or poor fMRI data quality													

a Abbreviations: MA, methamphetamine; MRI, magnetic resonance imaging; DTI, diffusion tensor imaging; MRS, magnetic resonance imaging; fMRI, functional MRI; HRT, hit response time; ISI, interstimulus interval; EPDS, Edinburgh postnatal depression scale; BDI, Beck depression inventory; FTT, failure to thrive; ADHD, attention deficit hyperactivity disorder; FSIQ, full scale intelligence quotient; HIV, human immunodeficiency virus; PCP, phencyclidine; LSD, lysergic acid diethylamide; alc, alcohol; nic/tob, nicotine/tobacco; marij, marijuana; cig, cigarette(s); AA, absolute alcohol; distrib, distribution; educ, education; soc/ec, socioeconomic. Dashes (−) indicate that no data were presented or the measure was not investigated. It should be noted that in the case of a discrepancy between the text of a reviewed article and the presented tables, we relied on the data in the (supplementary) tables.

b Special characteristics or exclusion criteria of the study groups are given in parentheses.

c Age at scanning, in the indicated units. Data are presented as mean ± SD.

d Gestational age at birth, in weeks. Data are presented as mean ± SD.

e *C*_head_ is the head circumference. Values are at birth unless otherwise noted. Data are presented as mean ± SD.

f Age at delivery, in years. Data are presented as mean ± SD.

g Years of education. Data are presented as mean ± SD.

h Depression, as measured by the indicated scale.

i Age at first scan.

j Age at second scan.

**Table 2:** Overview of the Reviewed Articles: Imaging Method, Between-Groups Brain Imaging Findings, and Associations with Cognitive and Behavioral Findings^a^

author	field strength (T)	method of analysis/toolbox	main findings	cognitive and behavioral characteristics of MA-exposed children relative to unexposed participants
Structural MRI Studies				
Chang et al., 2004^17^	1.5	morphometry/DEC-ALPHA	smaller bilateral putamen, globus pallidus, and hippocampus	poorer performance on the visual-motor integration task
			association between worse delayed verbal memory and smaller putamen and globus pallidus and between worse visual-motor integration score and smaller globus pallidus	worse performance on the sustained attention test
				significantly poorer long delay verbal memory and long delay spatial memory
Sowell et al., 2010^32^	1.5	tensor-based morphometry	smaller volumes of bilateral subcortical thalamic and striatal regions and larger volumes of limbic cortices	decreased full scale intelligence quotient
Derauf et al., 2012^33^	3	morphometry/FreeSurfer	smaller caudate	increased (worse) HRT by ISI scores in cognition evaluation tests
			no difference in cortical thickness	
			correlation between MA-exposure status, mean caudate volume, and HRT by ISI scores	
Roos et al., 2014^34^	3	morphometry/FreeSurfer	increased left putamen volume	–/–
			reduced left cortical thickness of the inferior parietal, parsopercularis, and precuneus areas	
			gender effects	
			boys: increased globus pallidus and bilateral diencephalon	
			girls: decreased midposterior corpus callosum	
Warton et al., 2018^29^	3	morphometry/FreeSurfer	correlation between MA exposure and less right caudate volume and decreased left caudate and bilateral thalamus volumes at a trend level	–/–
Roos et al., 2020^38^	3	graph analysis/FreeSurfer	no significant between-group difference in normalized characteristic path length and clustering coefficient left superior parietal region and striatal hubs had increased connectivity changes over time in the MA-exposed group	–/–
			similar patterns of change were observed in network connectivity on a regional level between groups	
			MA-exposed group had significantly less change in modularity and transitivity compared to the control group (segregation parameters)	
			network resilience as a measure of the minimum number of hubs needed to retain network integrity was not different between groups on a global or local level	
			decreased cortical thickness in left precentral region, right caudal middle frontal, and right rostral anterior cingulate and increased cortical thickness in left superior parietal region	
			decreased volume in left amygdala and increased volume in right putamen	
DTI Studies				
Cloak et al., 2009^11^	3	DTIStudio	decreased ADC in right frontal and bilateral parietal WM A trend for increased FA in the left frontal WM	–/–
Colby et al., 2012^30^	1.5	TBSS/FSL	increased FA in the genu of corpus callosum, left hemisphere internal and external capsule and corona radiata	decreased visual-motor integration score
			decreased MD and RD but increased AD in the lateral corona radiata	decreased IQ
Roos et al., 2015^10^	3	TBSS/FSL	fecreased FA and increased MD and RD in the tracts that crossed the striatal, limbic, and frontal regions	poorer performance on motor coordination and executive function tests
			correlation between decreased FA and poorer motor coordination	
Chang et al., 2016^28^	3	DtiStudio	in the superior corona radiata	decreased active muscle tone
			boys: decreased age-dependent changes of FA and increased diffusivity (MD, AD, and RD) at earlier postmenstrual age with sharper declines later	
			girls: no differences	
			in the posterior corona radiata	
			boys: increased diffusivity (MD, AD, and RD) at earlier postmenstrual age with sharper declines	
			girls: no differences	
			in the anterior corona radiata	
			boys: no differences	
			girls: decreased age-dependent changes of FA across time	
Warton et al., 2018^29^	3	tractography/AFNI	decreased FA in 3 WM connection: midbrain-left putamen, right putamen-right orbitofrontal cortex, and right putamen-right amygdala	–/–
			increased RD in midbrain-right caudate	
Warton et al. 2020^61^	3	probabilistic tractography/AFNI	negative association between MA exposure and FA in several WM connections in all five networks (commissural, left and right association, and left and right projection)	no difference in neonatal behavioral assessment scale
			positive association between MA exposure and RD in several WM connections in all five networks (commissural, left and right association, and left and right projection)	
MRS Studies				
Smith et al., 2001^24^	1.5	localized ^1^H-MRS/SPARC 2 workstation	increased total creatine in the striatum	social problems in 17%, aggressive behavior and anxious profile in 8% of MA-exposed children but no differences to unexposed participants
			a trend for decreased [NA/Cr] in the frontal WM	
Chang et al., 2009^35^	3	localized ^1^H-MRS/LCModel program	frontal WM: increased total creatine, N-acetyl compounds, and glutamate + glutamine but decreased myoinositol/total creatine	decreased performance on Beery-Visual Motor Integration
			thalamus: decreased myoinositol	
			decreased thalamic myoinositol was associated with poorer performance on the Beery-Visual Motor Integration	
			integration scores in both groups of children	
			increased frontal white matter CHO was associated with poorer performance on the Expressive One Word Picture Vocabulary test scores in all children	
fMRI Studies				
Lu et al., 2009^36^	3	BOLD/FSL	during the verbal memory task	decreased Full-Scale IQ
			increased activation in bilateral medial temporal, bilateral basal ganglia, and right occipito-temporal regions	decreased performance on California Verbal Learning Test
			lateralized activation of the left medial temporal region only in control group, which correlated to better performance	
Roussotte et al., 2011^18^	3	BOLD/FSL	during the visuospatial working memory task	decreased performance on the Visuospatial working memory task
			decreased activation in the frontal and basal ganglia regions in the left hemisphere	decreased full scale intelligence quotient
			especially in the MA-exposed group, performance was negatively correlated to the activation in the left parahippocampal gyrus, bilateral pre- and postcentral gyri, superior temporal gyrus, and putamen	
Roussotte et al., 2012^25^	3	seed-based functional connectivity analysis/FSL	decreased correlation between activity caudate seeds and prefrontal regions	decreased performance on the Visuospatial working memory task
			reduced functional connectivity between the dorsal caudate and frontal executive network	decreased full scale intelligence quotient
			increased functional connectivity between the posterior putamen and the frontal executive network	

a Abbreviations: MA, methamphetamine; MRI, magnetic resonance imaging; DTI, diffusion tensor imaging; MRS, magnetic resonance imaging; fMRI, functional MRI; HRT, hit response time; ISI, interstimulus interval; TBSS, tract-based spatial statistics; WM, white matter; FA, fractional anisotropy; MD, mean diffusivity; AD, axial diffusivity; RD, radial diffusivity; BOLD, blood-oxygen level-dependent.

## Results and Discussion

### Overview of the Included Studies

This Review aimed to summarize and discuss recent findings regarding alterations in brain imaging of offspring with prenatal exposure to MA. Our search resulted in 206 nonduplicate papers, which were screened for eligibility. After full-text screening, a total of 17 studies were selected. The studies employed four different imaging modalities, including structural MRI (*n* = 6), DTI (*n* = 6), MRS (*n* = 2), and fMRI (*n* = 3). However, it should be noted that some studies reported findings for the same study population using different modalities or analyses. In this regard, three structural MRI, DTI, and structural connectivity studies by Roos et al. in 2014,^34^ 2015,^10^ and 2020^38^ were conducted on the same cohort of 6–7 year-old children. Three structural MRI and DTI investigations by Warton et al. in 2018,^29^ 2018,^31^ and 2020^61^ were carried out on infants from a larger prospective longitudinal study of prenatal alcohol and drug exposure on infant development in South Africa. Lastly, two fMRI studies by Roussotte et al. in 2011^18^ and 2012^25^ were performed on the same groups of children.

The demographic and social/habitual characteristics of the mothers and offspring are presented in Table 1. The mean age of the participants varied significantly across studies with a range from infancy to 15 years. Of the 17 studies, four were conducted on infants and the rest were carried out on preschool and school children. Most of the included studies reported that mothers and offspring in MA-exposed and control groups had comparable gender, maternal age, offspring age, and gestational age. Regarding the education and IQ, mixed results were reported; while some studies included participants comparable based on education and IQ, other studies reported that the mothers of the MA-exposed group had a decreased IQ^28−30^ and a lower educational level compared to the mothers of the unexposed control group.^11,28,29,31^ Most of the studies revealed that a higher proportion of mothers of the MA-exposed groups smoked tobacco or consumed alcohol and marijuana during pregnancy compared to mothers in the control group.

Table 2 presents the studies exploring the effects of prenatal MA exposure on the offspring’s brain divided by imaging modality. Structural MRI studies used tensor-based morphometry, FreeSurfer, and graph theoretical analysis. The methods of analysis for DTI studies were tract-based spatial statistics (TBSS) and tractography. Both MRS studies used localized ^1^H-MRS analysis. The three fMRI studies were conducted during the performance of a task: two of them analyzed brain activity and the other explored functional connectivity with a seed-based approach.

In the following sections, we present and discuss imaging findings of MA-exposed offspring in distinct location-based categories of cortical and subcortical regions.

### Effects of Prenatal Exposure to MA on the Offspring’s Cortical Regions

**Figure 2:**
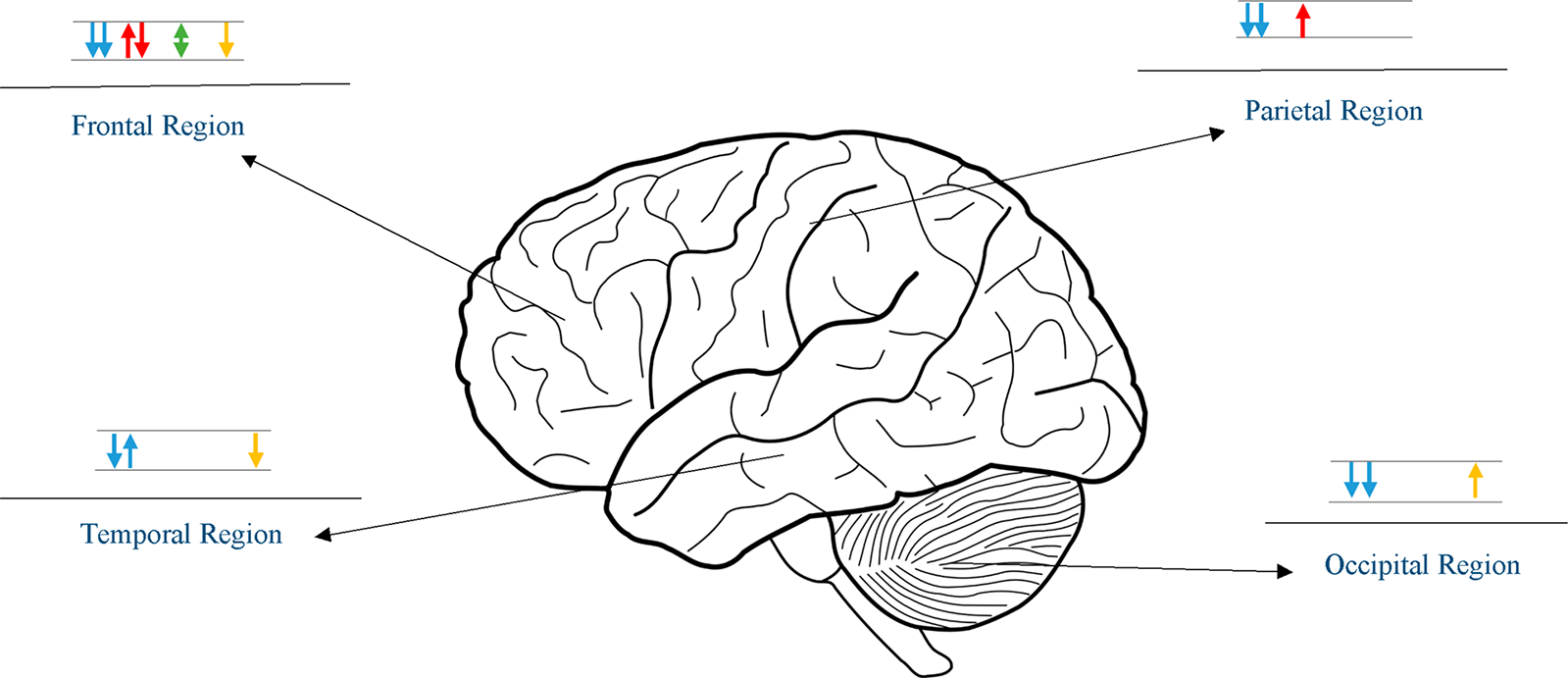
Illustration showing the most affected cortical regions in prenatal methamphetamine (MA) exposure. Each arrow represents a separate study. Upward blue arrow: increased volume/thickness; downward blue arrow: decreased volume/thickness; upward red arrow: increased microstructural integrity; downward red arrow: decreased microstructural integrity; green arrow: altered metabolite concentration; upward yellow arrow: increased functional activity/connectivity; downward yellow arrow: decreased functional activity/connectivity.

The included studies demonstrated that prenatal MA exposure is associated with both structural and functional changes across cortical regions, particularly in the frontal region (Figure 2). Structural MRI investigations revealed decreased volumes in left parieto-occipital and right anterior prefrontal cortices^32^ and in the posterior part of the superior temporal sulcus^33^ in children exposed to MA. In addition, decreased and increased volumes in ventral and medial temporal lobes and bilateral perisylvian cortices^32^ were also described. Moreover, Roos et al. reported significantly decreased left cortical thickness in the inferior parietal lobe, pars opercularis, and precuneus in the MA-exposed children; they also observed gender effects on volume differences between the two groups.^34^ A DTI study by Cloak et al., assessing WM microstructural integrity, reported that MA-exposed children had decreased apparent diffusion coefficient, mainly in the right frontal and bilateral parietal WM. They did not observe significant differences in FA between the two groups, but they reported a trend for increased FA in the left frontal WM in the MA-exposed group.^11^ Lastly, only one study assessed the association between brain structure and the neurocognitive profile of children; it showed a positive correlation between bilateral occipital volumes and full-scale intelligence quotient scores and a negative correlation between the volume of the left inferior temporal fusiform region and full-scale intelligence quotient scores.^32^

Alterations in the metabolic concentrations of cortical regions were limited to the frontal region. In a ^1^H-MRS study, Smith et al. did not report any significant differences in metabolite concentrations or ratios in the frontal WM but only a trend for decreased N-acetyl compounds/creatine in the frontal WM in the MA-exposed offspring.^24^ However, Chang et al. demonstrated that MA-exposed children had increased total creatine, N-acetyl compounds, and glutamate + glutamine and decreased myoinositol/total creatine in the frontal WM compared to the control group. They also reported increased choline compounds in the frontal WM of children with MA exposure throughout the pregnancy. Exploring the gender effects, they reported significantly decreased myoinositol/total creatine in MA-exposed girls but only a trend for decreased myoinositol/total creatine in MA-exposed boys in the frontal WM, and they explained these findings with a trend for the more significant elevation of total creatine and slightly decreased myoinositol in the MA-exposed girls. They also showed that increased N-acetyl compounds/total creatine in the frontal WM correlated to age in both MA-exposed and control groups.^35^

Changes in the functional activity and connectivity in MA-exposed offspring were investigated during verbal and working memory tasks and were distributed across the frontal, parietal, temporal, and occipital regions. Investigating the brain activity during a verbal memory task, Lu et al. found that the MA-exposed group showed increased activation in bilateral medial temporal and right occipitotemporal regions (along with basal ganglia) compared to the control group. In the control group, better verbal memory performances correlated with increased activation in the medial temporal regions.^36^ Roussotte et al. conducted two task-based fMRI studies on the same group of offspring exposed to prenatal MA. In the first study, they showed that, during a working memory task, inferior frontal gyrus showed decreased activation in the MA-exposed group compared to the control group. In both control and MA-exposed groups, activation in the inferior and middle temporal gyri, temporal pole, orbitofrontal cortex and frontal pole bilaterally, and the left superior frontal gyrus were negatively correlated with task accuracy. Especially in the MA-exposed group, activation in the left parahippocampal gyrus, bilateral pre- and postcentral gyri, and superior temporal gyrus was negatively associated with task performance.^18^ In the second study, they used a seed-based functional connectivity analysis with striatal nuclei as the seeds and demonstrated that cortical regions had altered functional connectivity with striatum in MA-exposed offspring (see below).^25^

### Effects of Prenatal Exposure to MA on the Offspring’s Subcortical Regions

#### Basal Ganglia

**Figure 3:**
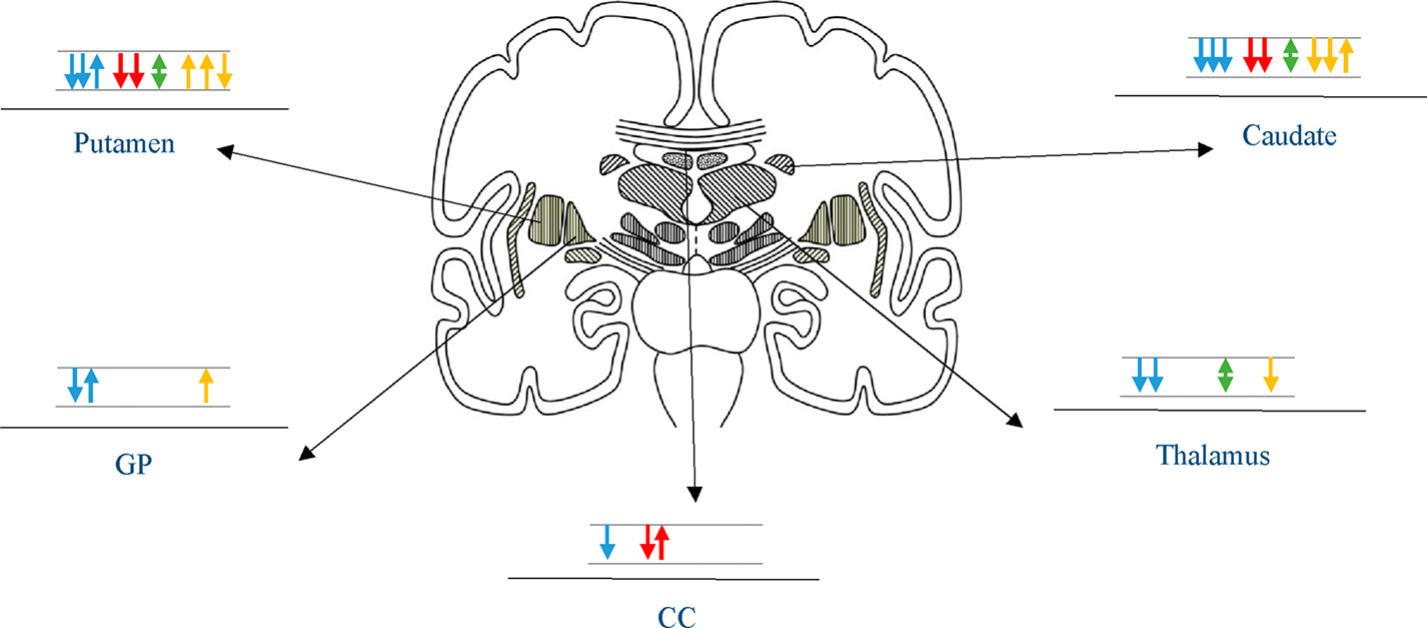
Illustration showing the most affected subcortical structures in prenatal methamphetamine (MA) exposure. Each arrow represents a separate study. Upward blue arrow: increased volume/thickness; downward blue arrow: decreased volume/thickness; upward red arrow: increased microstructural integrity; downward red arrow: decreased microstructural integrity; green arrow: altered metabolite concentration; upward yellow arrow: increased functional activity/connectivity; downward yellow arrow: decreased functional activity/connectivity.

Basal ganglia are a set of nuclei located in the subcortical region that are mainly involved in the regulation of movement and reward.^37^ Our review demonstrated that basal ganglia are the most affected structures in the MA-exposed offspring, probably in a wider circuit of fronto-thalamo-striatal connections (Figure 3). Of five volumetric studies, four showed reduced volumes of basal ganglia,^17,31−33^ and only one study reported increased volumes.^34^ Putamen,^17,32,38^ caudate,^32,33,31^ and pallidum^17^ were reported to have decreased volume in MA-exposed children. Differently, Roos et al.^34^ reported increased putamen volume in MA-exposed children compared to the control group and increased globus pallidus volume in MA-exposed males compared to male controls; they also observed gender effects on basal ganglia volume differences between the two groups: MA-exposed males had increased volumes of left globus pallidus and bilateral ventral diencephalon compared to control males.^34^ Regarding cortical thickness, one study showed no between-group difference^33^ but another study reported decreased cortical thickness in the left precentral region, right caudal middle frontal, and right rostral anterior cingulate and increased cortical thickness in the left superior parietal region.^38^ Alterations in striatal and pallidal volumes were associated with neurocognitive deficits in offspring. Two volumetric studies investigated children’s neurocognitive performance and showed that prenatal MA exposure was associated with deficits in visual-motor integration, verbal and spatial memory, attention, cognition, and mental development.^17,33^ Particularly, Chang et al. reported that impaired verbal memory was associated with smaller putamen and globus pallidus volumes and impaired visual-motor integration was associated with smaller globus pallidus volume.^17^ Accordingly, Derauf et al. showed a significant correlation between caudate volume and impaired attention in MA-exposed children.^33^

In line with volumetric studies, DTI research has shown that WM fibers connecting basal ganglia to other structures are affected by prenatal MA exposure. Tractography analysis on MA-exposed neonates demonstrated that the mean FA of fibers connecting midbrain to left putamen, right putamen to the right orbitofrontal cortex, and right putamen to the right amygdala is decreased in MA-exposed neonates.^29^ Moreover, RD was increased in the midbrain-right caudate connection in MA-exposed neonates.^29^ Furthermore, Roos et al. performed a longitudinal graph theoretical analysis to investigate the effects of prenatal MA exposure on the structural connectivity of brain networks in school-aged children. In the prenatal MA-exposed group, striatal hubs showed greater changes in connectivity over time. No significant between-group difference in the normalized characteristic path length and clustering coefficient was found. Similar patterns of change were observed in network connectivity on a regional level between groups. As they reported, the segregation of networks (modularity and transitivity of structural networks) showed less change in the MA-exposed group compared to the control group. They suggested that the observed increased striatal and also decreased frontal connectivity might result in increased risk-taking activity in prenatal MA-exposed children.^38^

Using ^1^H-MRS, Smith et al. conducted a study on 26 children (12 with a history of prenatal MA exposure and 14 control children) on the right frontal WM and right striatal voxels. They reported that the total creatinine was significantly increased in the striatum of MA-exposed children compared to the control group.^24^

All three task-based fMRI studies investigating the pattern of brain function in prenatal MA exposure demonstrated that basal ganglia are affected. Bilateral basal ganglia were demonstrated to have increased activation in MA-exposed children compared to controls during a verbal memory task.^36^ In the two task-based fMRI studies by Roussotte et al.,^18,25^ it was demonstrated that basal ganglia, particularly caudate and putamen, had decreased activation during working memory in MA-exposed children compared to the controls. In the MA-exposed group, the activity of putamen was negatively correlated with performance on the working memory task.^18^ Examining the same study group, with seed-based functional connectivity analysis, they investigated the functional connectivity between striatal seeds and other brain regions.^25^ They found a positive correlation between caudate seeds and prefrontal regions, which was more noticeable in the control group than MA-exposed ones. They reported a negative correlation between the caudate seeds with the cerebellum, occipital cortex, and bilateral primary motor cortex in the control group and fewer negatively correlated regions with caudate seeds in MA-exposed children. They also reported a negative correlation between the putamen seeds with the dorsolateral prefrontal cortex, the posterior cingulate, the precuneus, and the angular gyrus bilaterally in the control group and fewer negative correlations with superior frontal regions in the MA-exposed group. Additionally, they reported relatively reduced functional connectivity between the dorsal caudate and frontal executive network in the MA-exposed group. Parallel to their hypothesis, MA-exposed children had increased functional connectivity between the posterior putamen and the frontal executive network compared to the control group. These intriguing results corroborated their hypothesis that putamen might show increased connectivity with the frontal executive network as a compensatory response to damaged caudate and reduced connectivity of caudate with these frontal regions.^25^

**Figure 4:**
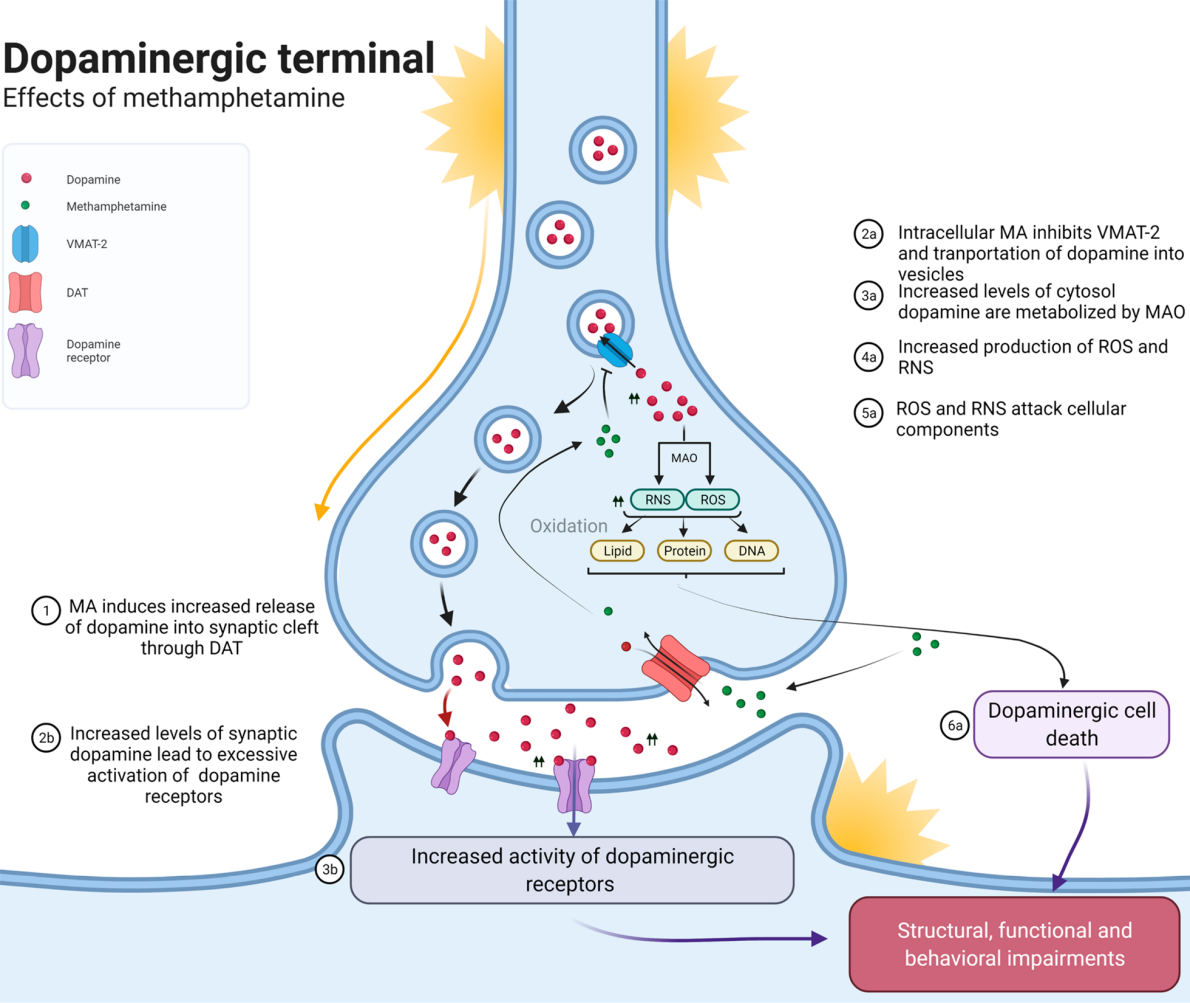
Illustration showing the underlying molecular mechanisms through which methamphetamine (MA) might affect brain structure and function.

The findings from reviewed neuroimaging studies showed that basal ganglia, and in particular striatum, are the most affected structures in offspring exposed to prenatal MA. However, the underlying molecular mechanisms through which MA exerts its neurotoxic effects cannot be presumed from neuroimaging studies in the Review. Nonetheless, findings from in vivo and in vitro studies suggest that oxidative stress due to dysregulation of dopaminergic metabolism and transmission is largely accountable for the detrimental effects of MA (Figure 4).^39−41^ MA exposure leads to excessive dopamine discharge into the synaptic space through dopamine transporter (DAT)-mediated inward transport of MA with simultaneous outward transport of dopamine.^42^ The ensuing increased levels of synaptic dopamine and thus elevated activation of dopamine receptors lead to most of the physical and psychological effects of MA, such as addiction and psychomotor dysregulation. In another distinct mechanism in dopaminergic terminals, MA inhibits the vesicular monoamine transporter 2 (VMAT-2), which is responsible for the sequestration of dopamine into vesicles. This, in turn, leads to increased dopamine levels in the cytosol, which is an oxidizing environment compared to vesicles where dopamine is normally stored.^43^ In the cytosol, dopamine is metabolized by monoamine oxidase-B (MAO-B), ultimately resulting in the production of reactive oxygen species (ROS) and reactive nitrogen species (RNS) through several reactions.^44−48^ These ROS and RNS cause oxidative damage on phospholipids, proteins, and nucleic acids, leading to dopaminergic cell death. In sum, basal ganglia structure and function seem to be altered in offspring of a mother who abused MA during pregnancy, presumably because of the effect of MA on the dopaminergic system. Interestingly, animal and human studies have shown that dopamine transporters are altered in offspring exposed prenatally to MA,^49^ which suggests the overstimulation of dopamine receptors in utero caused by dopamine overflow may result in an abnormal neurotransmitter activity threshold during adulthood.

#### Thalamus and Limbic Structures

The evidence shows that the thalamus and the limbic system are affected in MA-exposed offspring, both structurally and functionally. It was reported that the thalamus^31,32^ and hippocampus^17^ were smaller in MA-exposed offspring compared to the controls. It was also shown that the right thalamus volume was positively associated with full-scale intelligence quotient scores of MA-exposed children. Also, Chang et al. reported decreased myoinositol in the thalamus in the MA-exposed group.^35^ However, Sowell et al. showed an increased volume of anterior and posterior cingulate cortices in MA-exposed offspring.^32^ In agreement, DTI studies found that fornix and WM fibers connecting the orbitofrontal cortex and amygdala to putamen had decreased FA in MA-exposed offspring.^10,29^ In a longitudinal DTI study with graph theoretical analysis, multiple limbic hubs in the structural network had fewer changes in MA-exposed offspring compared to controls.^38^

Task-based fMRI studies also found that prenatal exposure to MA was associated with altered functional features in the thalamus and limbic system. Roussotte et al. reported that MA-exposed children had a decreased activation in the bilateral thalamus during working memory tasks compared to the control group. It was also observed that activations in anterior cingulate and paracingulate gyri and left parahippocampal gyrus were negatively correlated with task performance.^18^

The thalamus receives dopaminergic projections from the striatum and projects them to the frontal cortex.^50^ It is also involved in prenatal MA-exposed children’s brain alterations. Myoinositol was decreased in the thalamus of MA-exposed children, which is in line with the poorer performance of them in the visual-motor integration task.^35^ Results of a structural MRI study showed that prenatal MA exposure is associated with decreased bilateral thalamus at a trend level,^31^ which is in line with reduced thalamic gray matter in subjects addicted to alcohol, cannabis, nicotine, MA, cocaine, and opioids.^51−59^ Previous literature demonstrated reduced WM and gray matter integrity, baseline metabolism, and at rest, functional connectivity in the thalamus in drug abuser individuals.^59^ Taking together the thalamus as a central region in cortico-striato-thalamocortical circuit,^59,60^ showed reduced volume, decreased activation during working memory tasks and altered metabolite concentrations in the child with prenatal MA exposure relative to unexposed ones.

#### WM Fibers

WM fibers connecting bilateral regions or connecting higher level to lower level structures have also been demonstrated to have structural deficits in MA-exposed offspring. In particular, MA-exposed females presented decreased midposterior corpus callosum volume compared to control females.^34^ According to Colby et al., in the MA-exposed group, FA was significantly higher in the genu of the corpus callosum, left hemisphere internal and external capsules, and corona radiata compared to the control group. They also observed group effects in a region within the left anterior corona radiata. MD and RD were decreased, but AD in this area was increased in the MA-exposed group compared to the control group.^30^

Roos et al. demonstrated that, compared to the control group, MA-exposed children had significantly decreased FA in the left external capsule, fornix, and stria terminalis. Furthermore, they reported that, in these regions, MD and RD were increased in the MA-exposed children. Altered FA in these regions correlated with poorer performance in motor coordination and cognitive function in MA-exposed children, after controlling for confounding variables. In addition, there was a trend for decreased FA in the right external capsule to predict poorer motor coordination.^10^

Moreover, Warton et al.^61^ investigated the effects of prenatal MA exposure on the microstructure of global WM networks in neonates. Probabilistic tractography was used to estimate WM bundles associated with pairs of target regions within five networks (commissural fibers, left and right projection fibers, left and right association fibers). After controlling confounding variables, they showed negative associations between MA exposure and FA in several WM connections in all five networks, and positive associations were found between MA exposure and RD in several WM connections in all five networks. The increase in abnormal reflexes was associated with decreased FA in the left projection fiber network, but the performance on the neonatal behavioral assessment scale was not associated with FA in any of the other networks.

Chang et al. (2016), in a prospective longitudinal study, showed some sex-specific alterations in developmental age-dependent changes in MA-exposed infants. They reported that FA increased and diffusivity decreased with age in all participants. They demonstrated in the superior corona radiata, MA-exposed boys had decreased age-dependent changes of FA at an earlier age, which normalized at a later age. Also, they had increased diffusivity measures at earlier postmenstrual age but had sharper declines with age compared to control infants. At the same time, the girls did not show any differences between the two groups. Similarly, in the posterior corona radiata, diffusivity measures started increased and declined sharper in the MA-exposed boys, and no differences were seen between girl groups. In the anterior corona radiata in the MA-exposed girls, the age-dependent changes of FA remained decreased compared to unexposed girls across the time, but boys showed no differences. Additionally, they reported independent of sex, in the retrolenticular internal capsule, MA-exposed infants showed altered developmental age-dependent changes in AD compared to unexposed infants. They also evaluated all infants with Amiel-Tison Neurological Assessment at Term examination and showed that MA-exposed infants had poorer active muscle tones and increased total score, indicating poorer function, which normalized after three to four months after birth.^28^

Cloak et al. demonstrated that children exposed to MA had a reduced apparent diffusion coefficient in the right frontal and right and left parietal WM compared to the control children.^11^ Similarly, another study reported decreased MD and RD in lateral corona radiata in MA-exposed children,^30^ but conversely, Roos et al. reported increased MD and RD in the left external capsule, fornix, and stria terminalis in the MA-exposed children.^10^ In line with Roos et al., another study reported increased RD in the midbrain-right caudate connection in the MA-exposed infants relative to the control group.^29^ Additionally, the results of these studies were inconsistent in the comparison of FA between groups. Colby et al.^30^ reported increased FA, while Roos et al.^10^ reported decreased FA in the left external capsule and Warton et al.^29,61^ reported decreased FA in the three WM connections (midbrain-left putamen, right putamen-right orbitofrontal cortex, and right putamen-right amygdala) in the MA-exposed children. These inconsistencies might be due to the heterogeneity of the participants in terms of age,^10^ which ranged from infancy to school age. Moreover, differences in methods and selecting regions of interest might cause this discrepancy.

### Sex-Specific Alterations

MA-exposed girls had decreased midposterior corpus callosum volume relative to control girls, but MA-exposed boys had relatively increased left globus pallidus and right and left ventral diencephalon.^34^ Another study investigated sex-specific alterations in developmental age-dependent changes in MA-exposed children and found that MA-exposed boys had decreased age-dependent changes of FA in the superior corona radiata compared to the control boys, which normalized at a later age. Diffusivity in the superior and posterior corona radiata of MA-exposed boys started to increase but declined more sharply relative to unexposed boys, but no differences were seen between girl groups in these regions. Normalization of the age-dependent changes of FA and diffusivity in later postmenstrual age, compared to the early years, might have occurred because of neuronal repair or other structural changes occurring as a compensatory mechanism to the MA-induced neuronal damage after cessation of the MA exposure. Conversely, in the MA-exposed girls, in the anterior corona radiata, the age-dependent changes of FA remained decreased relative to control girls across time, while boys showed no differences.^28^ Considering that MA can inhibit the dopamine transporter at body temperature^49,62^ and that previous investigations on rats have revealed a sexual dimorphism of the dopamine transporter system,^49^ we might speculate that the toxic effect of MA on the dopaminergic system varies in the two sexes.

### Neurocognition and Behavioral Alterations

Preschool and school MA-exposed children showed poorer performances on visual-motor integration tasks,^17,28^ and also, they had a decreased score on sustained attention, delayed verbal memory, and delayed spatial memory tests.^17^ Moreover, MA-exposed children had significantly decreased Full Scale Intelligence Quotient (FSIQ)^32^ and poorer sustained attention.^33^ In a cohort study, children with prenatal MA exposure showed increased aggressive behavior, and children with MA exposure throughout pregnancy showed more aggressive behavior than those who were exposed in only one trimester. These results are in line with studies on adult MA abusers^63^ that show a wide range of cognitive impairments, including executive functions, attention, working memory, impulsivity, and social cognition. In sum, the included investigations showed that MA-exposed children presented decreased cognitive performances in comparison with healthy controls, which might be due to the neural toxicity of MA during brain development and also the deprived socio-economical context in which the MA-exposed children are raised.^64^

### Strengths and Limitations

The divergence in the methods of analysis and the selection of regions of interest were important limitations to compare the results of the reviewed studies. The use of different methods of analysis across studies made it difficult to quantitatively compare the results. Thus, we herein could only compare the result qualitatively. Another considerable limitation was polysubstance exposure, especially alcohol and tobacco, as a confounding variable; to address this issue, some studies tried to exclude children whose mother abused other drugs than MA,^28,38^ some used multivariable analyses to control this issue,^10,18,31,35^ and some included a separate group who used alcohol but did not use MA^30^ to differentiate their effect on the brain. One noteworthy strength of this study was the consistent results of some regions from the different studies with different modalities, which confirmed each other.

### Clinical and Nonclinical Implications and Further Direction of the Studies

To confirm the results of these studies, future studies with more participants should investigate how alterations in brain macrostructural, microstructural, metabolic, and functional characteristics might mediate the association between prenatal MA exposure and related neurocognitive deficits. A confirmed interpretation of structural, metabolic, and functional alterations in the brain of prenatal MA-exposed children would help clinicians to diagnose earlier and choose more specific therapeutic targets to prevent and treat the developmental disorders. Future studies should evaluate possible pharmacological and cognitive treatments to improve these children’s functional and social performance.

## Conclusion

In this study, we systematically reviewed macrostructural, microstructural, metabolic, and functional brain abnormalities in children exposed to prenatal MA. Studies have used different MRI modalities (including conventional MRI, DTI, MRS, and fMRI) and different methods of analysis to investigate the effect of prenatal MA exposure on brain development. Our findings demonstrated that prenatal MA exposure was associated with macrostructural, microstructural, metabolic, and functional deficits in both cortical and subcortical areas. However, the most affected regions were striatal nuclei, frontal region, thalamus and the limbic system, and WM fibers connecting these regions. The findings from our study might have valuable implications for targeted treatment of neurocognitive and behavioral deficits in children with prenatal MA exposure. Even so, our results should be interpreted cautiously due to the heterogeneity of the included studies in terms of study populations and methods of analysis.

## References

[ref1] Fleckenstein A. E.; Volz T. J.; Riddle E. L.; Gibb J. W.; Hanson G. R. (2007) Annu. Rev. Pharmacol. Toxicol. 47, 681–698. 10.1146/annurev.pharmtox.47.120505.105140. 17209801

[ref2] Sambo D. O.; Lebowitz J. J.; Khoshbouei H. (2018) The sigma-1 receptor as a regulator of dopamine neurotransmission: A potential therapeutic target for methamphetamine addiction. Pharmacol. Ther. 186, 152–167. 10.1016/j.pharmthera.2018.01.009. 29360540 PMC5962385

[ref3] Yang W.; Yang R.; Luo J.; He L.; Liu J.; Zhang J. (2018) Increased Absolute Glutamate Concentrations and Glutamate-to-Creatine Ratios in Patients With Methamphetamine Use Disorders. Frontiers in psychiatry 9, 368 10.3389/fpsyt.2018.00368. 30233420 PMC6128240

[ref4] Padgett C. L.; Lalive A. L.; Tan K. R.; Terunuma M.; Munoz M. B.; Pangalos M. N.; Martínez-Hernández J.; Watanabe M.; Moss S. J.; Luján R.; Lüscher C.; Slesinger P. A. (2012) Methamphetamine-evoked depression of GABA(B) receptor signaling in GABA neurons of the VTA. Neuron 73, 978–989. 10.1016/j.neuron.2011.12.031. 22405207 PMC3560416

[ref5] Cruickshank C. C.; Dyer K. R. (2009) A review of the clinical pharmacology of methamphetamine. Addiction 104, 1085–1099. 10.1111/j.1360-0443.2009.02564.x. 19426289

[ref6] (2006) Methamphetamine use and HIV risk behaviors among heterosexual men--preliminary results from five northern California counties, December 2001-November 2003. Morb. Mortal. Wkly. Rep. 55, 273. 16543881

[ref7] Good M. M.; Solt I.; Acuna J. G.; Rotmensch S.; Kim M. J. (2010) Methamphetamine use during pregnancy: maternal and neonatal implications. Obstet. Gynecol. 116, 330–334. 10.1097/AOG.0b013e3181e67094. 20664393

[ref8] Winslow B. T.; Voorhees K. I.; Pehl K. A. (2007) Methamphetamine abuse. Am. Fam. Physician 76, 1169–1174. 17990840

[ref9] Terplan M.; Smith E. J.; Kozloski M. J.; Pollack H. A. (2009) Methamphetamine use among pregnant women. Obstet. Gynecol. 113, 1285–1291. 10.1097/AOG.0b013e3181a5ec6f. 19461424

[ref10] Roos A.; Kwiatkowski M. A.; Fouche J.-P.; Narr K. L.; Thomas K. G.; Stein D. J.; Donald K. A. (2015) White matter integrity and cognitive performance in children with prenatal methamphetamine exposure. Behav. Brain Res. 279, 62–67. 10.1016/j.bbr.2014.11.005. 25446763 PMC7188961

[ref11] Cloak C. C.; Ernst T.; Fujii L.; Hedemark B.; Chang L. (2009) Lower diffusion in white matter of children with prenatal methamphetamine exposure. Neurology 72, 2068–2075. 10.1212/01.wnl.0000346516.49126.20. 19369643 PMC2697962

[ref12] Kalaitzopoulos D.-R.; Chatzistergiou K.; Amylidi A.-L.; Kokkinidis D. G.; Goulis D. G. (2018) Effect of methamphetamine hydrochloride on pregnancy outcome: a systematic review and meta-analysis. J. Addict. Med. 12, 220–226. 10.1097/ADM.0000000000000391. 29509557

[ref13] Smith L. M.; LaGasse L. L.; Derauf C.; Grant P.; Shah R.; Arria A.; Huestis M.; Haning W.; Strauss A.; Della Grotta S.; et al. (2006) The infant development, environment, and lifestyle study: effects of prenatal methamphetamine exposure, polydrug exposure, and poverty on intrauterine growth. Pediatrics 118, 1149–1156. 10.1542/peds.2005-2564. 16951010

[ref14] Diaz S. D.; Smith L. M.; LaGasse L. L.; Derauf C.; Newman E.; Shah R.; Arria A.; Huestis M. A.; Della Grotta S.; Dansereau L. M.; et al. (2014) Effects of prenatal methamphetamine exposure on behavioral and cognitive findings at 7.5 years of age. J. Pediatr. 164, 1333–1338. 10.1016/j.jpeds.2014.01.053. 24630350 PMC4035384

[ref15] Piper B. J.; Acevedo S. F.; Kolchugina G. K.; Butler R. W.; Corbett S. M.; Honeycutt E. B.; Craytor M. J.; Raber J. (2011) Abnormalities in parentally rated executive function in methamphetamine/polysubstance exposed children. Pharmacol., Biochem. Behav. 98, 432–439. 10.1016/j.pbb.2011.02.013. 21334365 PMC3069661

[ref16] LaGasse L. L.; Wouldes T.; Newman E.; Smith L. M.; Shah R. Z.; Derauf C.; Huestis M. A.; Arria A. M.; Della Grotta S.; Wilcox T. (2011) Prenatal methamphetamine exposure and neonatal neurobehavioral outcome in the USA and New Zealand. Neurotoxicol. Teratol. 33, 166–175. 10.1016/j.ntt.2010.06.009. 20615464 PMC2974956

[ref17] Chang L.; Smith L. M.; LoPresti C.; Yonekura M. L.; Kuo J.; Walot I.; Ernst T. (2004) Smaller subcortical volumes and cognitive deficits in children with prenatal methamphetamine exposure. Psychiatry Res., Neuroimaging 132, 95–106. 10.1016/j.pscychresns.2004.06.004. 15598544

[ref18] Roussotte F. F.; Bramen J. E.; Nunez S. C.; Quandt L. C.; Smith L.; O’Connor M. J.; Bookheimer S. Y.; Sowell E. R. (2011) Abnormal brain activation during working memory in children with prenatal exposure to drugs of abuse: the effects of methamphetamine, alcohol, and polydrug exposure. NeuroImage 54, 3067–3075. 10.1016/j.neuroimage.2010.10.072. 21040792 PMC4405109

[ref19] O’Donnell L. J.; Westin C. F. (2011) An introduction to diffusion tensor image analysis. Neurosurg Clin N Am. 22, 185–196. 10.1016/j.nec.2010.12.004. 21435570 PMC3163395

[ref20] Ghazi Sherbaf F.; Aarabi M. H.; Hosein Yazdi M.; Haghshomar M. (2019) White matter microstructure in fetal alcohol spectrum disorders: A systematic review of diffusion tensor imaging studies. Human Brain Mapping 40, 1017–1036. 10.1002/hbm.24409. 30289588 PMC6865781

[ref21] Assaf Y.; Pasternak O. (2008) Diffusion tensor imaging (DTI)-based white matter mapping in brain research: a review. J. Mol. Neurosci. 34, 51–61. 10.1007/s12031-007-0029-0. 18157658

[ref22] Aung W. Y.; Mar S.; Benzinger T. L. (2013) Diffusion tensor MRI as a biomarker in axonal and myelin damage. Imaging Med. 5, 427–440. 10.2217/iim.13.49. 24795779 PMC4004089

[ref23] Tromp D. (2016) The diffusion tensor, and its relation to FA, MD, AD and RD. Winnower 8, e146119.94804 10.15200/winn.146119.94804.

[ref24] Smith L. M.; Chang L.; Yonekura M. L.; Grob C.; Osborn D.; Ernst T. (2001) Brain proton magnetic resonance spectroscopy in children exposed to methamphetamine in utero. Neurology 57, 255–260. 10.1212/WNL.57.2.255. 11468309

[ref25] Roussotte F. F.; Rudie J. D.; Smith L.; O’Connor M. J.; Bookheimer S. Y.; Narr K. L.; Sowell E. R. (2012) Frontostriatal connectivity in children during working memory and the effects of prenatal methamphetamine, alcohol, and polydrug exposure. Dev. Neurosci. 34, 43–57. 10.1159/000336242. 22472800

[ref26] Moher D.; Liberati A.; Tetzlaff J.; Altman D. G. (2009) Preferred reporting items for systematic reviews and meta-analyses: the PRISMA statement. PLoS Med. 6, e1000097 10.1371/journal.pmed.1000097. 19621072 PMC2707599

[ref27] Liberati A.; Altman D. G.; Tetzlaff J.; Mulrow C.; Gotzsche P. C.; Ioannidis J. P.; Clarke M.; Devereaux P. J.; Kleijnen J.; Moher D. (2009) The PRISMA statement for reporting systematic reviews and meta-analyses of studies that evaluate health care interventions: explanation and elaboration. PLoS Med. 6, e1000100 10.1371/journal.pmed.1000100. 19621070 PMC2707010

[ref28] Chang L.; Oishi K.; Skranes J.; Buchthal S.; Cunningham E.; Yamakawa R.; Hayama S.; Jiang C. S.; Alicata D.; Hernandez A.; et al. (2016) Sex-specific alterations of white matter developmental trajectories in infants with prenatal exposure to methamphetamine and tobacco. JAMA psychiatry 73, 1217–1227. 10.1001/jamapsychiatry.2016.2794. 27829078 PMC6467201

[ref29] Warton F. L.; Taylor P. A.; Warton C. M.; Molteno C. D.; Wintermark P.; Lindinger N. M.; Zöllei L.; van der Kouwe A.; Jacobson J. L.; Jacobson S. W.; et al. (2018) Prenatal methamphetamine exposure is associated with corticostriatal white matter changes in neonates. Metab. Brain Dis. 33, 507–522. 10.1007/s11011-017-0135-9. 29063448 PMC5866741

[ref30] Colby J. B.; Smith L.; O’Connor M. J.; Bookheimer S. Y.; Van Horn J. D.; Sowell E. R. (2012) White matter microstructural alterations in children with prenatal methamphetamine/polydrug exposure. Psychiatry Res., Neuroimaging 204, 140–148. 10.1016/j.pscychresns.2012.04.017. PMC3634917 23149028

[ref31] Warton F. L.; Meintjes E. M.; Warton C. M. R.; Molteno C. D.; Lindinger N. M.; Carter R. C.; Zöllei L.; Wintermark P.; Jacobson J. L.; van der Kouwe A.; Jacobson S. W. (2018) Prenatal methamphetamine exposure is associated with reduced subcortical volumes in neonates. Neurotoxicol. Teratol. 65, 51–59. 10.1016/j.ntt.2017.10.005. 29069607 PMC5803390

[ref32] Sowell E. R.; Leow A. D.; Bookheimer S. Y.; Smith L. M.; O’Connor M. J.; Kan E.; Rosso C.; Houston S.; Dinov I. D.; Thompson P. M. (2010) Differentiating prenatal exposure to methamphetamine and alcohol versus alcohol and not methamphetamine using tensor-based brain morphometry and discriminant analysis. J. Neurosci. 30, 3876–3885. 10.1523/JNEUROSCI.4967-09.2010. 20237258 PMC2847574

[ref33] Derauf C.; Lester B. M.; Neyzi N.; Kekatpure M.; Gracia L.; Davis J.; Kallianpur K.; Efird J. T.; Kosofsky B. (2012) Subcortical and cortical structural central nervous system changes and attention processing deficits in preschool-aged children with prenatal methamphetamine and tobacco exposure. Dev. Neurosci. 34, 327–341. 10.1159/000341119. 22907274 PMC4091037

[ref34] Roos A.; Jones G.; Howells F. M.; Stein D. J.; Donald K. A. (2014) Structural brain changes in prenatal methamphetamine-exposed children. Metab. Brain Dis. 29, 341–349. 10.1007/s11011-014-9500-0. 24553878

[ref35] Chang L.; Cloak C.; Jiang C.; Farnham S.; Tokeshi B.; Buchthal S.; Hedemark B.; Smith L.; Ernst T. (2009) Altered neurometabolites and motor integration in children exposed to methamphetamine in utero. NeuroImage 48, 391–397. 10.1016/j.neuroimage.2009.06.062. 19576287 PMC3142567

[ref36] Lu L. H.; Johnson A.; O’Hare E. D.; Bookheimer S. Y.; Smith L. M.; O’Connor M. J.; Sowell E. R. (2009) Effects of prenatal methamphetamine exposure on verbal memory revealed with functional magnetic resonance imaging. J. Dev Behav Pediatr 30, 185–192. 10.1097/DBP.0b013e3181a7ee6b. 19525715 PMC2745202

[ref37] Kim T.; Hamade K. C.; Todorov D.; Barnett W. H.; Capps R. A.; Latash E. M.; Markin S. N.; Rybak I. A.; Molkov Y. I. (2017) Reward Based Motor Adaptation Mediated by Basal Ganglia. Front. Comput. Neurosci. 11, 19 10.3389/fncom.2017.00019. 28408878 PMC5374212

[ref38] Roos A.; Fouche J. P.; du Toit S.; du Plessis S.; Stein D. J.; Donald K. A. (2020) Structural brain network development in children following prenatal methamphetamine exposure. J. Comp. Neurol. 528, 1856–1863. 10.1002/cne.24858. 31953852

[ref39] Johnson J. A.; Johnson D. A.; Kraft A. D.; Calkins M. J.; Jakel R. J.; Vargas M. R.; Chen P. C. (2008) The Nrf2-ARE pathway: an indicator and modulator of oxidative stress in neurodegeneration. Ann. N. Y. Acad. Sci. 1147, 61–69. 10.1196/annals.1427.036. 19076431 PMC2605641

[ref40] Krasnova I. N.; Cadet J. L. (2009) Methamphetamine toxicity and messengers of death. Brain Res. Rev. 60, 379–407. 10.1016/j.brainresrev.2009.03.002. 19328213 PMC2731235

[ref41] Ares-Santos S.; Granado N.; Moratalla R. (2013) The role of dopamine receptors in the neurotoxicity of methamphetamine. J. Intern. Med. 273, 437–453. 10.1111/joim.12049. 23600399

[ref42] Liang N. Y.; Rutledge C. O. (1982) Evidence for carrier-mediated efflux of dopamine from corpus striatum. Biochem. Pharmacol. 31, 2479–2484. 10.1016/0006-2952(82)90057-0. 7126258

[ref43] Hansen J. P.; Riddle E. L.; Sandoval V.; Brown J. M.; Gibb J. W.; Hanson G. R.; Fleckenstein A. E. (2002) Methylenedioxymethamphetamine decreases plasmalemmal and vesicular dopamine transport: mechanisms and implications for neurotoxicity. J. Pharmacol. Exp. Ther. 300, 1093–1100. 10.1124/jpet.300.3.1093. 11861820

[ref44] Ali S. F.; Imam S. Z.; Itzhak Y. (2005) Role of peroxynitrite in methamphetamine-induced dopaminergic neurodegeneration and neuroprotection by antioxidants and selective NOS inhibitors. Ann. N. Y. Acad. Sci. 1053, 97–98. 10.1111/j.1749-6632.2005.tb00014.x. 16179512

[ref45] Beckman J. S.; Koppenol W. H. (1996) Nitric oxide, superoxide, and peroxynitrite: the good, the bad, and ugly. American journal of physiology 271, C1424–1437. 10.1152/ajpcell.1996.271.5.C1424. 8944624

[ref46] Cadet J. L.; Brannock C. (1998) Free radicals and the pathobiology of brain dopamine systems. Neurochem. Int. 32, 117–131. 10.1016/S0197-0186(97)00031-4. 9542724

[ref47] Imam S. Z.; Newport G. D.; Duhart H. M.; Islam F.; Slikker W. Jr.; Ali S. F. (2002) Methamphetamine-induced dopaminergic neurotoxicity and production of peroxynitrite are potentiated in nerve growth factor differentiated pheochromocytoma 12 cells. Ann. N. Y. Acad. Sci. 965, 204–213. 10.1111/j.1749-6632.2002.tb04162.x. 12105096

[ref48] Imam S. Z.; Newport G. D.; Itzhak Y.; Cadet J. L.; Islam F.; Slikker W. Jr.; Ali S. F. (2001) Peroxynitrite plays a role in methamphetamine-induced dopaminergic neurotoxicity: evidence from mice lacking neuronal nitric oxide synthase gene or overexpressing copper-zinc superoxide dismutase. J. Neurochem. 76, 745–749. 10.1046/j.1471-4159.2001.00029.x. 11158245

[ref49] Sirova J.; Kristofikova Z.; Vrajova M.; Fujakova-Lipski M.; Ripova D.; Klaschka J.; Slamberova R. (2016) Sex-Dependent Changes in Striatal Dopamine Transport in Preadolescent Rats Exposed Prenatally and/or Postnatally to Methamphetamine. Neurochem. Res. 41, 1911–1923. 10.1007/s11064-016-1902-4. 27038442

[ref50] Haber S.; Mcfarland N. R. (2001) The place of the thalamus in frontal cortical-basal ganglia circuits. Neuroscientist 7, 315–324. 10.1177/107385840100700408. 11488397

[ref51] Chanraud S.; Martelli C.; Delain F.; Kostogianni N.; Douaud G.; Aubin H.-J.; Reynaud M.; Martinot J.-L. (2007) Brain Morphometry and Cognitive Performance in Detoxified Alcohol-Dependents with Preserved Psychosocial Functioning. Neuropsychopharmacology 32, 429–438. 10.1038/sj.npp.1301219. 17047671

[ref52] Mon A.; Durazzo T. C.; Abe C.; Gazdzinski S.; Pennington D.; Schmidt T.; Meyerhoff D. J. (2014) Structural brain differences in alcohol-dependent individuals with and without comorbid substance dependence. Drug Alcohol Depend. 144, 170–177. 10.1016/j.drugalcdep.2014.09.010. 25263262 PMC4280666

[ref53] Hanlon C. A.; Owens M. M.; Joseph J. E.; Zhu X.; George M. S.; Brady K. T.; Hartwell K. J. (2016) Lower subcortical gray matter volume in both younger smokers and established smokers relative to non-smokers. Addiction biology 21, 185–195. 10.1111/adb.12171. 25125263 PMC4326619

[ref54] Morales A.; Kohno M.; Robertson C. L.; Dean A. C.; Mandelkern M. A.; London E. D. (2015) Gray-matter volume, midbrain dopamine D2/D3 receptors and drug craving in methamphetamine users. Mol. Psychiatry 20, 764–771. 10.1038/mp.2015.47. 25896164 PMC4440838

[ref55] Sim M. E.; Lyoo I. K.; Streeter C. C.; Covell J.; Sarid-Segal O.; Ciraulo D. A.; Kim M. J.; Kaufman M. J.; Yurgelun-Todd D. A.; Renshaw P. F. (2007) Cerebellar gray matter volume correlates with duration of cocaine use in cocaine-dependent subjects. Neuropsychopharmacology 32, 2229–2237. 10.1038/sj.npp.1301346. 17299505

[ref56] Mashhoon Y.; Sava S.; Sneider J.; Nickerson L.; Silveri M. (2015) Cortical thinness and volume differences associated with marijuana abuse in emerging adults. Drug Alcohol Depend. 155, 275–283. 10.1016/j.drugalcdep.2015.06.016. 26249265 PMC4581973

[ref57] Liao Y.; Tang J.; Liu T.; Chen X.; Hao W. (2012) Differences between smokers and non-smokers in regional gray matter volumes: a voxel-based morphometry study. Addiction biology 17, 977–980. 10.1111/j.1369-1600.2010.00250.x. 20731627

[ref58] Ide J. S.; Zhang S.; Hu S.; Sinha R.; Mazure C. M.; Li C.-s. R. (2014) Cerebral gray matter volumes and low-frequency fluctuation of BOLD signals in cocaine dependence: duration of use and gender difference. Drug Alcohol Depend. 134, 51–62. 10.1016/j.drugalcdep.2013.09.004. 24090712 PMC3865077

[ref59] Huang A. S.; Mitchell J. A.; Haber S. N.; Alia-Klein N.; Goldstein R. Z. (2018) The thalamus in drug addiction: from rodents to humans. Philos. Trans. R. Soc., B 373, 20170028 10.1098/rstb.2017.0028. PMC5790826 29352027

[ref60] Haber S. N.; Knutson B. (2010) The reward circuit: linking primate anatomy and human imaging. Neuropsychopharmacology 35, 4–26. 10.1038/npp.2009.129. 19812543 PMC3055449

[ref61] Warton F. L.; Taylor P. A.; Warton C. M. R.; Molteno C. D.; Wintermark P.; Zöllei L.; van der Kouwe A. J.; Jacobson J. L.; Jacobson S. W.; Meintjes E. M. (2020) Reduced fractional anisotropy in projection, association, and commissural fiber networks in neonates with prenatal methamphetamine exposure. Dev. Neurobiol. 80, 381–398. 10.1002/dneu.22784. 33010114 PMC7855045

[ref62] Torres G. E.; Gainetdinov R. R.; Caron M. G. (2003) Plasma membrane monoamine transporters: structure, regulation and function. Nat. Rev. Neurosci. 4, 13–25. 10.1038/nrn1008. 12511858

[ref63] Mizoguchi H.; Yamada K. (2019) Methamphetamine use causes cognitive impairment and altered decision-making. Neurochem. Int. 124, 106–113. 10.1016/j.neuint.2018.12.019. 30611760

[ref64] Dinger J.; Hinner P.; Reichert J.; Rüdiger M. (2017) Methamphetamine Consumption during Pregnancy - Effects on Child Health. Pharmacopsychiatry 50, 107–113. 10.1055/s-0042-122711. 28178739

